# Acute effects of postural changes and lower body positive and negative pressure on the eye

**DOI:** 10.3389/fphys.2022.933450

**Published:** 2022-08-31

**Authors:** M. P. Van Akin, O. M. Lantz, A. M. Fellows, Christine Toutain-Kidd, Michael Zegans, J. C. Buckey, A. P. Anderson

**Affiliations:** ^1^ University of Colorado Boulder, Ann and H.J. Smead Department of Aerospace Engineering Sciences, Boulder, CO, United States; ^2^ Geisel School of Medicine at Dartmouth College, Hanover, NH, United States

**Keywords:** spaceflight associated neuro-ocular syndrome, fluid shift, gravitational physiology, spaceflight analogue, ocular measures

## Abstract

**Introduction:** Entry into weightlessness results in a fluid shift and a loss of hydrostatic gradients. These factors are believed to affect the eye and contribute to the ocular changes that occur in space. We measured eye parameters during fluid shifts produced by lower body negative pressure (LBNP) and lower body positive pressure (LBPP) and changes in hydrostatic gradient direction (supine-prone) in normal subjects to assess the relative effects of fluid shifts and hydrostatic gradient changes on the eye.

**Methods:** Ocular parameters (intraocular pressure (IOP), ocular geometry, and optical coherence tomography measures) were measured in the seated, supine, and prone positions. To create a fluid shift in the supine and prone positions, the lower body chamber pressure ranged from -40 mmHg to +40 mmHg. Subjects maintained each posture and LBNP/LBPP combination for 15 min prior to data collection. A linear mixed-effects model was used to determine the effects of fluid shifts (as reflected by LBNP/LBPP) and hydrostatic gradient changes (as reflected by the change from seated to supine and from seated to prone) on eye parameters.

**Results:** Chamber pressure was positively correlated with both increased choroidal thickness (*β* = 0.11 , *p* = 0.01) and IOP (*β* = 0.06 *p* < 0.001). The change in posture increased IOP compared to seated IOP (supine *β* = 2.1, *p* = 0.01, prone *β* = 9.5, *p* < 0.001 prone) but not choroidal thickness. IOP changes correlated with axial length (R = 0.72, *p* < 0.001).

**Discussion:** The effects of hydrostatic gradients and fluids shifts on the eye were investigated by inducing a fluid shift in both the supine and prone postures. Both hydrostatic gradients (posture) and fluid shifts (chamber pressure) affected IOP, but only hydrostatic gradients affected axial length and aqueous depth. Changes in choroidal thickness were only significant for the fluid shifts. Changes in hydrostatic gradients can produce significant changes in both IOP and axial length. Fluid shifts are often cited as important factors in the pathophysiology of SANS, but the local loss of hydrostatic gradients in the head may also play an important role in these ocular findings.

## Introduction

A collection of physiological and pathologic neuro-ocular responses to long duration spaceflight affects between 38 and 51% of astronauts. (Stenger and Tarver, 2017; Lee et al., 2020) The space flight associated neuro-ocular syndrome (SANS) is defined clinically by the presence of optic disc edema (Stenger et al., 2019), but there are other associated ocular responses including globe flattening, retinal thickness changes, choroidal and retinal folds, and hyperopic refractive error shifts (Lee et al., 2017). These vision problems, if they are not correctable or progress, may jeopardize deep space mission objectives and crew safety (Stenger and Tarver, 2017; Lee et al., 2020). SANS is hypothesized to be tied to the duration of exposure, as ocular changes identified for space shuttle astronauts were transient, while SANS symptoms typically don’t develop until 3 weeks or more into flights. (Mader et al., 2011) SANS does not affect all astronauts suggesting individual difference play a role. The pathophysiology of SANS is currently unknown (Lee et al., 2017), but seems to be a unique effect of long-duration microgravity exposure. (Lee et al., 2017)

The cephalad fluid redistribution that occurs in microgravity has caused some to theorize a significant increase in intracranial pressure (ICP) may be present (Stenger and Tarver, 2017), such as occurs in idiopathic intracranial hypertension. To date, no direct ICP measurements have been made during prolonged spaceflight to confirm or disprove this hypothesis (Stenger and Tarver, 2017; Lee et al., 2020). Some findings of SANS, including optic disc edema and globe flattening, are consistent with this elevated ICP theory. (Mader et al., 2011) The magnitude of other findings, such as optic nerve sheath diameter increase, cannot entirely be explained by elevated ICP. (Shinojima et al., 2018) An upward shift of the brain and optic chiasm along with brain rotation around the edge of the cerebellar tentorium observed in postflight long duration astronauts, as well as findings that the optic nerve shifts forward using pre- and post-flight imaging, indicate that a greater understanding of the interplay between ICP and spaceflight relevant factors are needed. (Roberts et al., 2017; Shinojima et al., 2018; Wåhlin et al., 2021) Direct ICP measurements in parabolic flight, however, suggest ICP is not elevated in microgravity beyond Earth supine levels. (Lawley et al., 2017) Further, the presence of clinically elevated ICP (>20mmHg (Fernando et al., 2019)) is considered unlikely since invasively-measured central venous pressure also goes down in microgravity compared to supine values (Buckey et al., 1996). Recent bed rest studies suggest that a mild but chronic elevation in ICP compared to 24-h average ICP levels could induce ocular findings similar to those seen in spaceflight (Laurie et al., 2020), suggesting this could potentially lead to the SANS findings seen during long duration spaceflight. (Laurie et al., 2020) Cephalad fluid shifts from microgravity have also been documented to cause jugular vein distension and thickening of the retinal nerve fiber layer (RNFL) of the optic nerve (Mader et al., 2011). Some have suggested, these fluid shifts might lead to choroidal engorgement observed during long duration spaceflight (Stenger et al., 2019). Choroidal engorgement has also been observed in acute, ground-based studies (Anderson et al., 2016), but not in 30-days bed rest (Laurie et al., 2020). These effects of cephalad fluid shifts on cerebral and ocular hemodynamics may cause ocular changes during spaceflight.

The removal of hydrostatic gradients in microgravity may affect the contribution of gravitational loading to SANS findings. The fluid pressure in the eye, which is partially dependent on hydrostatic gradients, provides a counterforce opposing cerebral spinal fluid pressures at the back of the eye transmitted through the optic nerve head, resulting in a translaminar pressure. Based on findings from acute bed rest, the gravitational vector appears to play a role in the distribution of cerebrospinal fluid in the optic nerve sheath, which may cause SANS structural changes. (Marshall-Goebel et al., 2017)

The effect of gravitational loading on the body, manifested as both the weight of the body’s tissues and the direction of hydrostatic gradients, is another mechanism that should be considered when examining the pathophysiology of SANS. (Buckey et al., 2018) On Earth, the weight of tissue compresses the walls of vessels in the body. The loss of tissue weight and removal of the tissue compressive forces in microgravity may exacerbate the effect of fluid redistributions on astronauts. In support of this, astronaut weight, waist circumference, and chest circumference was significantly greater for those that developed optic disc edema and choroidal folds in weightlessness compared to those who did not (Buckey et al., 2018), suggesting that tissue weight, body shape, and distribution of tissue mass play a role in the development of SANS. Although it is not possible to remove the forces of tissue weight on Earth, the directionality of tissue weight loads can be altered with postural changes terrestrially to study this potential mechanism. Body weight is used as a proxy measurement of tissue weight. Although the anatomic distribution of body weight may also affect SANS findings, this is much more difficult to measure.

While moving from seated to supine and from seated to microgravity both change hydrostatic gradients and produce fluid shifts, the removal of tissue weight and hydrostatic gradients in all axes is unique to microgravity. Therefore, both changes of tissue weight and hydrostatic gradients must be studied in conjunction with fluid shifts as potential contributors to SANS. To understand these contributing mechanisms, this research investigates the independent effects of both fluid shifts and changes in the direction of gravity acting on the body, eye, and cardiovascular system to provide insight into the mechanisms of SANS. Fluid shifts and gravity-induced pressure gradients are coupled on Earth, where the gravity vector determines the direction of fluid shift as well as the direction of tissue compression and hydrostatic gradient. For example, on Earth, transitioning from seated to supine postures induces a fluid shift, and the hydrostatic gradient within the eye from the cornea to the retina creates a pressure at the retina. (Hata et al., 2012) In the prone position, the direction of this gradient is reversed, but there is no additional significant gravitationally-induced fluid shift compared to the supine position. (Savaser et al., 2013) The direction of tissue compression also changes in this posture. Thus, by making measurements in supine and prone positions, the effect of hydrostatic gradients and tissue weight can be isolated from the effect of fluid shifts. (Savaser et al., 2013) Previous studies have documented the acute effects of the gravity vector on the eye with a change in posture from seated baseline to supine and prone. (Anderson et al., 2016; Nelson et al., 2020) To investigate the effects of fluid shifts independent of posture, a fluid shift can be induced terrestrially using lower body positive (LBPP) and lower body negative pressure (LBNP). (Hirsch et al., 1989) Therefore, to decouple and study these effects, ocular and cardiovascular measurements in supine and prone, as well as in LBNP, LBPP, and lower body atmospheric pressure conditions were taken. Previous LBNP studies showed that 35.3 mmHg in the supine position created a ground reaction force equal to one body weight, but 20–25 mmHg was sufficient in reducing ICP. (Petersen et al., 2019)

Understanding the acute effects of fluid shifts and gravity-induced pressure gradients on the eye is important because they represent a perturbation from baseline as would be expected in spaceflight, and likely lead to the ocular changes observed after prolonged microgravity exposure. We hypothesized that both posture and lower body pressure changes would affect intraocular pressure (IOP), while only posture would affect axial length, aqueous depth, and choroidal thickness based on previous studies (Macias et al., 2015; Anderson et al., 2016). We hypothesized that tissue weight would affect IOP and mean arterial blood pressure (Lam et al., 2017). Corneal thickness, retinal nerve fiber layer thickness, and minimum rim width were opportunistically measured without *a priori* hypotheses.

## Methods

### Subjects

All human subject protocols were approved by the Committee for the Protection of Human Subjects at Dartmouth College. Informed consent was obtained from all subjects prior to the study. Measurements were recorded for 15 subjects (7 male, 8 female, age 27 ± 3). Subjects were in generally good health and were screened for ocular and systemic diseases. All subjects had no contraindications to LBNP/LBPP exposure, as assessed by a stand test to screen for cardiovascular responses, resting systolic blood pressure between 100–140 mmHg, and resting diastolic blood pressure between 70–90 mmHg. All subjects were screened to have an IOP below 20mmHg in the seated position. Subjects were studied at least 1 h after a light meal and asked to maintain euhydration the 24 h preceding data collection. Subjects refrained from alcohol, caffeine, and heavy exercise for 12 h before the trial.

### Independent variables

Measurements were recorded in a seated (baseline), supine, prone, prone with LBNP, prone with LBPP, supine with LBNP, and supine with LBPP. The order of these conditions was randomized for each subject. Positive pressure was maintained between 35 and 40 mmHg, and negative pressure was maintained between -35 and -40 mmHg. Not all subjects completed all postural and pressure conditions. Due to individual responses to each condition and time constraints, prone LBNP was the condition that was most often excluded. The number of test subjects that had data taken in each experimental condition are shown in Table 1.

**TABLE 1:** Number of test subjects by condition.

	Seated	Supine Atm	Supine with LBPP	Supine with LBNP	Prone atm	Prone with LBPP	Prone LBNP
Number of Test Subjects	15	15	15	13	14	14	10

### Dependent variables

IOP measurements were made using a Perkins tonometer by a trained ophthalmologic technician. Subject corneas were anesthetized using Fluorescein Sodium (0.25%) and benoxinate hydrochloride (0.4%).

A Heidelberg Spectralis Optical Coherence Tomography (OCT) was used to measure perifoveal choroidal thickness from raster scans. Choroidal thickness was measured by two independent researchers blinded to the experimental condition. Choroidal thickness was measured from the perceived choroid-sclera boundary to the posterior of the retinal pigment epithelium. One measurement was made in the scan closest to the fovea. The perifoveal choroidal thickness measurements recorded by the two observers had a correlation coefficient of R > 0.9, so the results were averaged to obtain a single value. Peripapillary RNFL thickness and minimum rim width (MRW) were taken from OCT glaucoma scans. These two metrics were measured by taking the average of a circular scan with a 12^o^ scan angle. RNFL thickness measurements were taken at a 3.5 mm diameter circle lefted at the optic disc.

Axial length, anterior chamber depth, and corneal thickness were measured using an optical biometer (Lenstar 900; Haag-Streit). Five replicates were averaged to obtain a single accurate geometric measurement.

Blood pressure and heart rate (HR) were continuously monitored using a Biopac Non-Invasive Blood Pressure Amplifier to ensure test subjects did not reach presyncope. Systolic and diastolic blood pressures were measured from a blood pressure cuff on the brachial artery. Mean arterial blood pressure (MAP) was calculated by adding 2/3 the quantity of the diastolic blood pressure to 1/3 the quantity of systolic blood pressure.

### Experimental design

**FIGURE 1:**
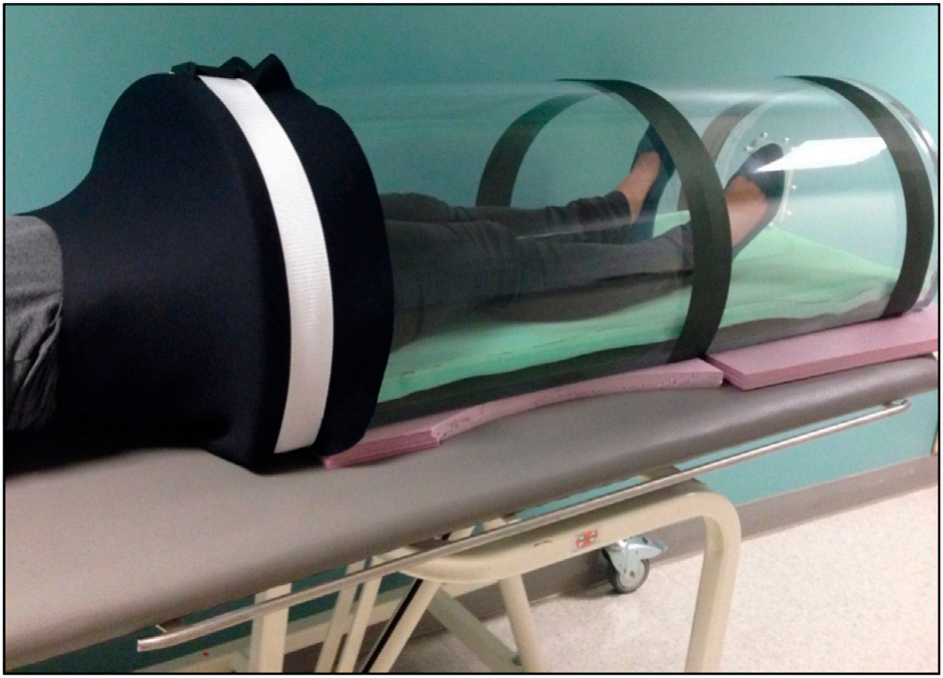
Dartmouth LBNP/LBPP device with neoprene seal.

This experiment was conducted in the Space Medicine Innovations Lab at the Giesel School of Medicine at Dartmouth College. Subject’s seated baseline was collected upon entering the laboratory. All subsequent experimental conditions were ordered randomly for each subject. Upon entering a new posture or lower body pressure condition, a 15-min waiting interval prior to data collection was used to achieve approximate homeostasis. This interval was selected to be larger than the time constants of ocular measures that respond to posture changes exponentially (Anderson et al., 2017). LBNP/LBPP was administered using a custom-built chamber. The seal between the subject and the chamber was achieved using a neoprene skirt worn by the subject and was positioned approximately at the iliac crest (Figure 1). The optical biometer and OCT device were mounted on a custom-built stand (Anderson et al., 2016) that allowed rotation for measurements to be made in the supine and prone postures.

### Statistical analysis

A linear mixed effects model analysis was performed to determine the effects of posture and fluid shift interventions on the eye and cardiovascular system. Seated measurements made without any lower body pressure were treated as a baseline, and all fixed effects are defined relative to the baseline condition. Chamber pressure, the supine posture, and the prone posture were modelled as fixed effects and subjects were modelled as random effects. The fixed effect of chamber pressure was modelled using a continuous pressure variable, while posture (supine or prone) was treated as a nominal variable. A fixed effect of subject weight was initially included in the model but subsequently removed because the effect was only significant for MAP. Therefore, the model presented does not have the fixed effect for weight. All dependent variable measures (e.g., IOP, axial length, etc.) were checked for outliers, homoscedasticity, and normality prior to implementing a linear mixed effects model. Outliers were assessed by studying residual plots. Subjects whose residuals statistically deviated from normality were considered for removal. Homoscedasticity was assessed by calculating the Spearman Rank correlation of the absolute residuals against the raw values of each measure. A Shapiro-Wilk test was performed on the residuals to test for normality. Pearson correlation coefficients were calculated between baseline normalized dependent variables of interest.

## Results

**FIGURE 2:**
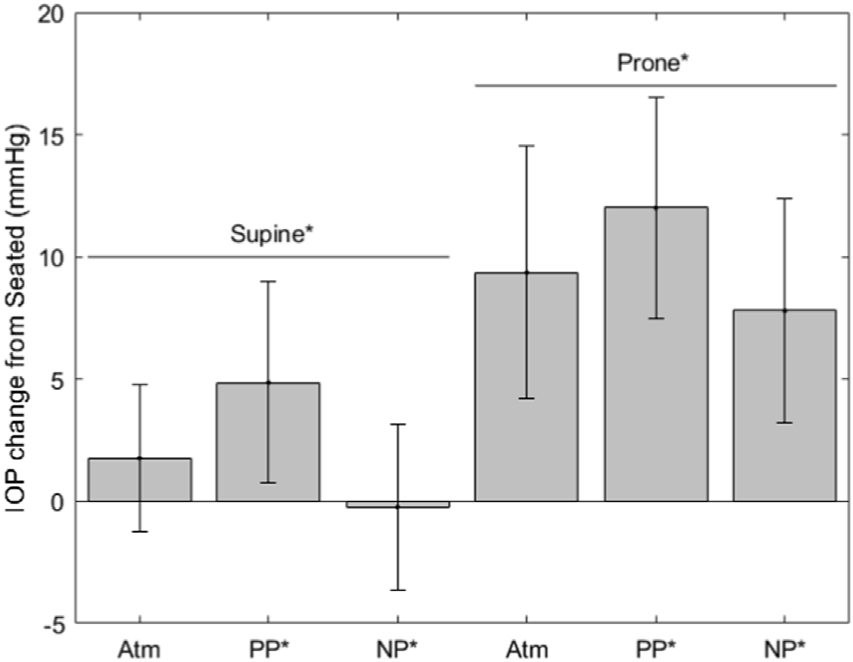
IOP change from seated measurements (* indicates significance, error bars indicate standard deviation, Atm = no lower body pressure, PP = lower body positive pressure, NP = lower body negative pressure).

**FIGURE 3:**
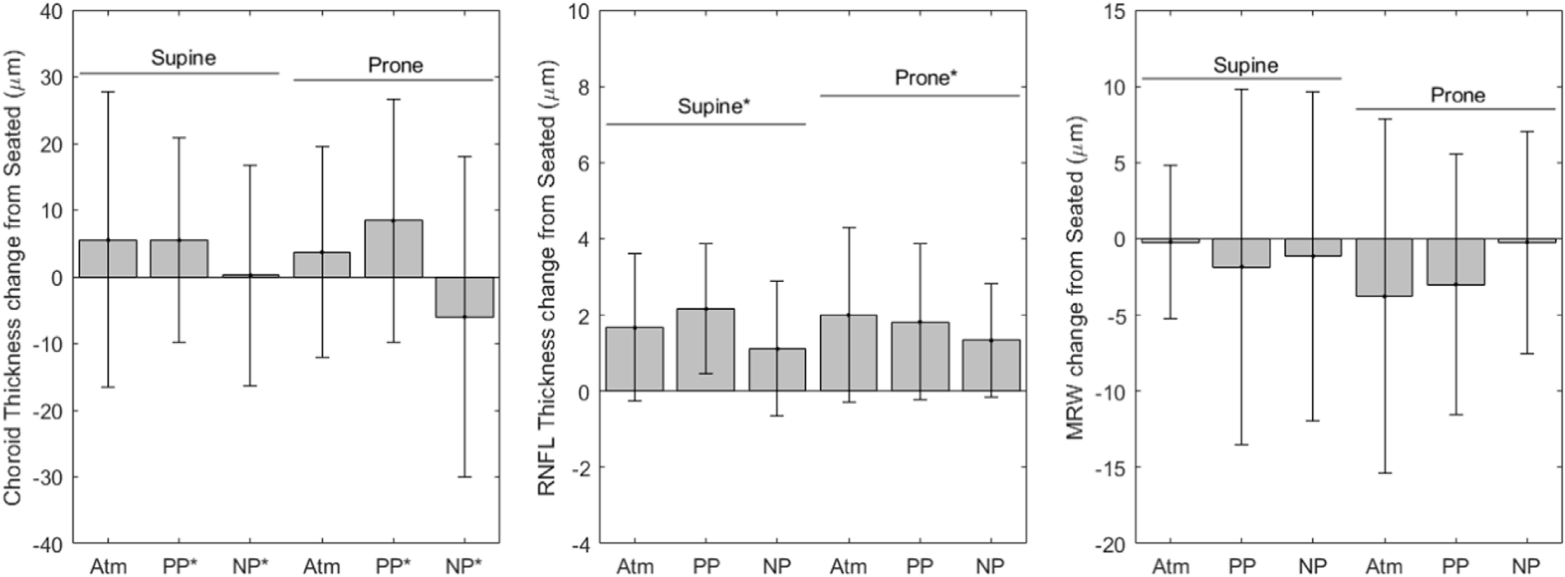
OCT change from seated measurements (* indicates significance, error bars indicate standard deviation, Atm = no lower body pressure, PP = lower body positive pressure, NP = lower body negative pressure).

**FIGURE 4:**
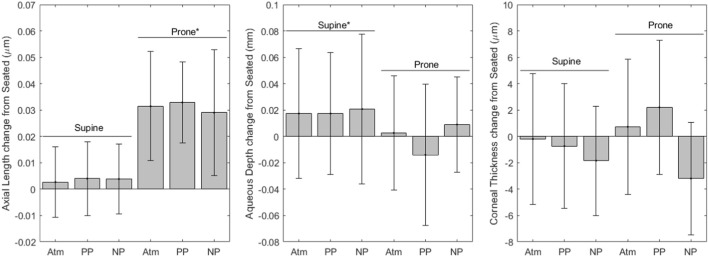
Optical Biometer change from seated measurements (* indicates significance, error bars indicate standard deviation, Atm = no lower body pressure, PP = lower body positive pressure, NP = lower body negative pressure).

**FIGURE 5:**
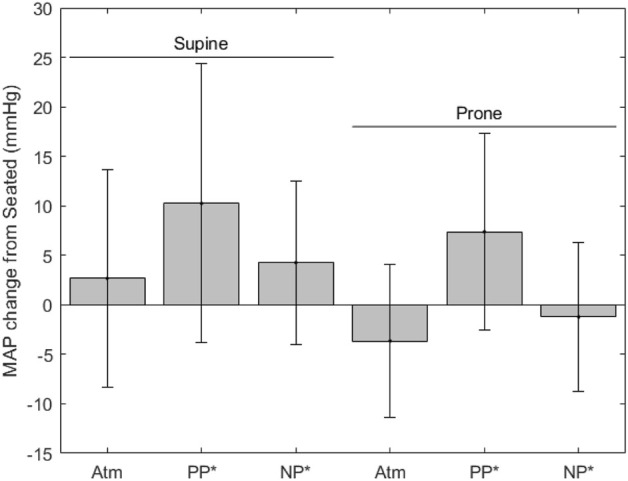
MAP change from seated measurements (* indicates significance, error bars indicate standard deviation, Atm = no lower body pressure, PP = lower body positive pressure, NP = lower body negative pressure).

Figures 2–5 show the average change of our subject’s change from the seated baseline in each condition, with error bars that demonstrate the standard deviation. Table 2 shows the fixed effects coefficients for each of the linear mixed effects model by dependent variable measures, with the statistically significant (*α* = 0.05) coefficients bolded. The fixed effect for subject weight in the linear mixed effects model was not significant for IOP but was for MAP.

**TABLE 2:** Fixed effects coefficients and confidence intervals (CI) for linear mixed effects models.

	β_pressure_ (units/mmHg)		β_supine_		β_prone_	
Ocular measure	Estimate	CI	Estimate	CI	Estimate	CI
IOP (mmHg)	**0.06**	**0.040–0.080**	**2.1**	**0.42–3.8**	**9.5**	**7.8–11**
Axial Length (mm)	4.1E-5	−4.1E-5–1.3E-4	0.0035	−0.0036–0.011	**0.031**	**0.024–0.038**
Aqueous Depth (mm)	−1.5E-4	−3.5E-4–5.6E-5	**0.019**	**0.0021–0.037**	−0.0027	−0.020–0.015
Corneal Thickness (µm)	0.029	0.0049–0.053	−0.73	−2.8–1.3	0.35	−1.7–2.4
Choroidal Thickness ( $\sqrt[{3}]{\text{µm}}$ )	**0.11**	**0.024–0.20**	3.5	−4.0–11	3	−4.5–10
RNFL Thickness (µm)	0.0065	-0.0068–0.020	**2.3**	**1.1–3.4**	**2.4**	**1.2–3.5**
MRW (µm)	−0.027	−0.080–0.026	−0.91	−5.4–3.6	−2.2	−6.8–2.4
MAP (mmHg)	**0.11**	**0.054–0.16**	5.2	0.70–9.7	0.82	−3.7–5.4

Statistically significant estimates at *p* < 0.05 in bold. Choroidal thickness coefficients are shown after the cube root transformation was applied.

The IOP measurements are shown in Figure 2. IOP values for each condition are as follows (mean ± SD, mmHg): seated, 14.6 ± 3.0; supine without LBNP/LBPP, 16.4 ± 3.0; supine with LBPP, 19.5 ± 3.9; supine with LBNP, 14.3 ± 3.5; prone without LBNP/LBPP, 23.9 ± 4.7; prone with LBPP, 26.6 ± 3.7; prone with LBNP, 22.7 ± 2.8. Significant effects were found for chamber pressure (*p* < 0.001), the supine posture (*p* < 0.02), and the prone posture (*p* < 0.001).

Figure 3 shows variables derived from OCT measurements. Choroidal thickness values for each condition are as follows (mean ± SD, µm): seated, 340.1 ± 109.1; supine without LBNP/LBPP, 345.6 ± 103.0; supine with LBPP, 345.6 ± 107.1; supine with LBNP, 333.9 ± 106.6; prone without LBNP/LBPP, 343.8 ± 110.7; prone with LBPP, 348.5 ± 111.5; prone with LBNP, 304.0 ± 99.0. The choroidal thickness data failed to meet the normality and homoscedasticity assumptions. A cube root transformation was applied to meet these assumptions for linear mixed effects models. A significant effect was found for pressure (*p* < 0.03), but not for the supine posture (*p* = 0.2) nor the prone posture (*p* = 0.4).

RNFL thickness values for each condition are as follows (mean ± SD, µm): seated, 96.25 ± 7.96; supine without LBNP/LBPP, 97.9 ± 7.01; supine with LBPP, 98.6 ± 7.11; supine with LBNP, 99.3 ± 7.54; prone without LBNP/LBPP, 98.2 ± 7.23; prone with LBPP, 98.6 ± 7.38; prone with LBNP, 98.0 ± 9.35. After the removal of two outliers, the RNFL data met the normality and homoscedasticity assumptions. Significant effects were found for the supine (*p* < 0.001) and prone postures (*p* < 0.001), but not for chamber pressure (*p* = 0.1). To ensure our results were not dependent only on the removal of outliers, the model was refit including all data points. The model coefficients were slightly changed, but all factors remained unchanged (i.e., supine and prone postures were statistically significant, but not chamber pressure).

The MRW values for each condition are as follows (mean ± SD, µm): seated, 362 ± 49; supine without LBNP/LBPP, 362 ± 50; supine with LBPP, 361 ± 44; supine with LBNP, 370 ± 44; prone without LBNP/LBPP, 359 ± 48; prone with LBPP, 359 ± 49; prone with LBNP, 370 ± 49. The MRW data were not normally distributed nor homoscedastic, even after transformations were applied. No alternative analysis methods were available due to missing subject data. We included the MRW model fixed-effects coefficients in Table 2 for completeness, although the values may not be reliable. No fixed-effects were significant (*p* > 0.05).

Figure 4 shows variables derived from optical biometer measurements. Axial length values for each condition are as follows (mean ± SD, mm): seated, 24.3 ± 1.5; supine without LBNP/LBPP, 24.3 ± 1.5; supine with LBPP, 24.3 ± 1.5; supine with LBNP, 24.3 ± 1.6; prone without LBNP/LBPP, 24.3 ± 1.5; prone with LBPP, 24.3 ± 1.5; prone with LBNP, 24.7 ± 1.5. After the removal of two outliers, the axial length data met the normality and homoscedasticity assumptions. Significant effects were found for the prone posture (*p* < 0.001), but not for pressure (*p* = 0.6) nor the supine posture (*p* = 0.2). To ensure our results were not dependent only on the removal of outliers, the model was refit including all data points. The model coefficients were slightly changed, but all factors remained unchanged (i.e., the prone posture were statistically significant, but not the supine posture or chamber pressure).

Aqueous depth values for each condition are as follows (mean ± SD, mm): seated, 3.22 ± 0.27; supine without LBNP/LBPP, 3.24 ± 0.25; supine with LBPP, 3.24 ± 0.27; supine with LBNP, 3.26 ± 0.27; prone without LBNP/LBPP, 3.22 ± 0.27; prone with LBPP, 3.21 ± 0.27; prone with LBNP, 3.27 ± 0.30. A significant effect was found for the supine posture (*p* < 0.03), but not for chamber pressure (*p* = 0.2) nor the prone posture (*p* = 0.8).

Corneal thickness values for each condition are as follows (mean ± SD, µm): seated, 549.1 ± 33.5; supine without LBNP/LBPP, 548.9 ± 31.7; supine with LBPP, 548.4 ± 33.5; supine with LBNP, 548.5 ± 33.6; prone without LBNP/LBPP, 549.9 ± 34.8; prone with LBPP, 551.3 ± 33.7; prone with LBNP, 548.3 ± 39.9. A significant effect was found for chamber pressure (*p* < 0.02), but not for the supine posture (*p* = 0.5) nor the prone posture (*p* = 0.7).

Figure 5 shows MAP, where values for each condition are as follows (mean ± SD, mmHg): seated, 90.4 ± 6.3; supine without LBNP/LBPP, 92.8 ± 7.5; supine with LBPP, 100.5 ± 13.0; supine with LBNP, 93.8 ± 8.5; prone without LBNP/LBPP, 87.3 ± 8.6 prone with LBPP, 98.2 ± 8.9; prone with LBNP, 90.1 ± 9.3. Significant effects were found for chamber pressure (*p* < 0.001) and the supine posture (*p* < 0.03), but not for the prone posture (*p* = 0.7).

HR values for each condition are as follows (mean ± SD, mmHg): seated, 65.4 ± 11.3; supine without LBNP/LBPP, 62.0 ± 9.8; supine with LBPP, 64.4 ± 9.5; supine with LBNP, 70.6 ± 12.4; prone without LBNP/LBPP, 65.7 ± 8.4 prone with LBPP, 71.5 ± 12.0; prone with LBNP, 71.2 ± 9.5. HR was not included in the linear mixed effects model.

**FIGURE 6:**
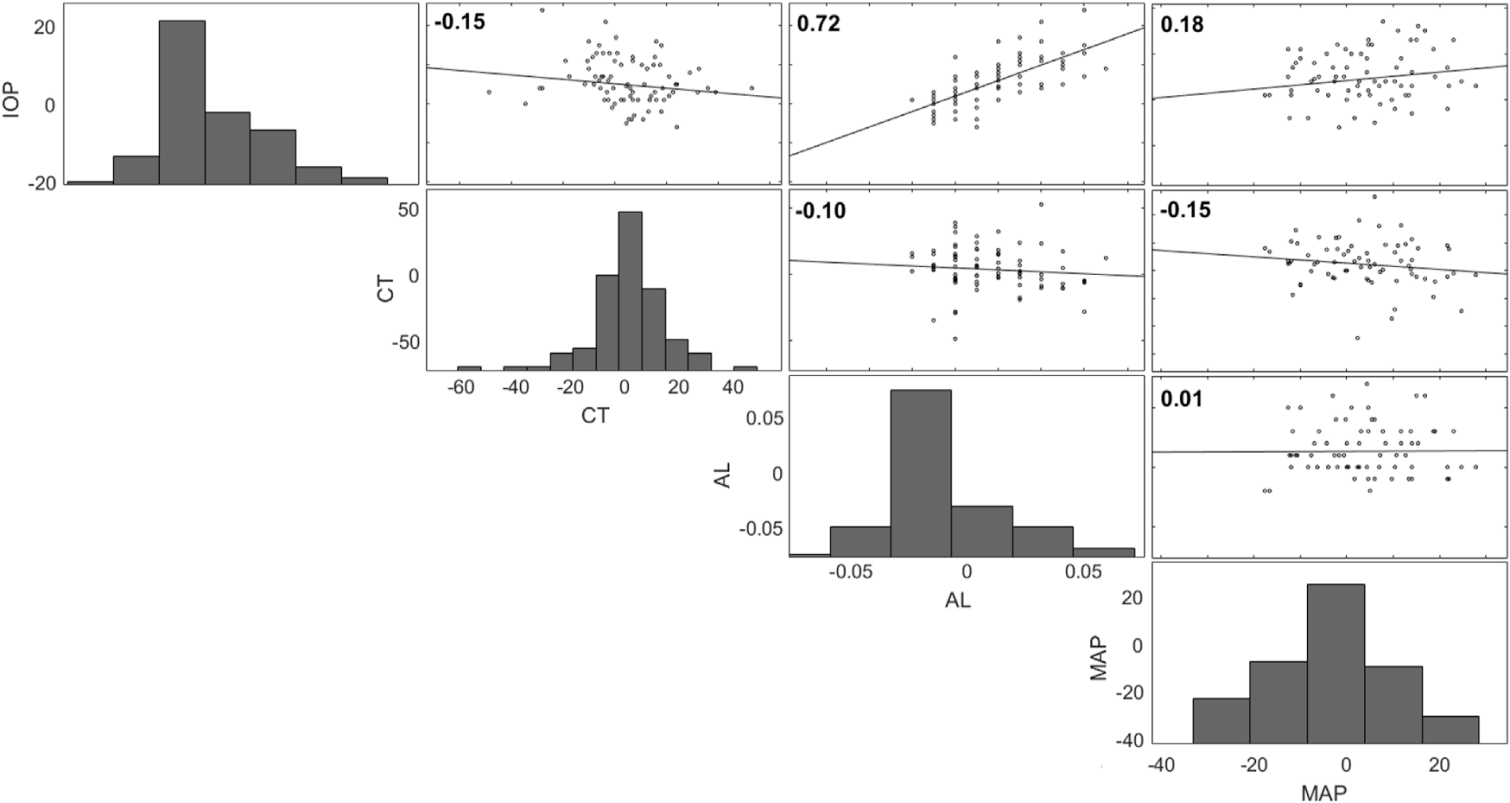
Correlation matrix for IOP, choroidal thickness (CT), axial length (AL), and MAP (all changes from seated). Each dependent variable is plotted on every one of the axes along the row or column that the respective histogram is plotted.

The calculated Pearson correlations between IOP and axial length, IOP and choroidal thickness, choroidal thickness and axial length, and IOP and MAP are shown in the top left of each plot in Figure 6. The data presented in Figure 6 are changes from the seated baseline values. Each dependent variable is plotted on every one of the axes along the row or column that the respective histogram is plotted. A significant result was found between IOP and axial length (R = 0.7, *p* < 0.001). The correlation was not significant between IOP and choroidal thickness (R = -0.1, *p* = 0.1), choroidal thickness and axial length (R = -0.06, *p* = 0.5) nor between IOP and MAP (R = 0.2, *p* = 0.1).

## Discussion

Our study demonstrates the acute effects of both fluid shifts and gravitational loading on the eye. IOP, choroidal thickness, and MAP were all significantly affected by fluid shifts in our study, as induced by changing lower body pressure. IOP, axial length, aqueous depth, and RNFL thickness were affected by gravitational loading, as altered by changing posture. Both fluid shifts and gravitational loading acutely affect the geometry and pressures in the eye, although the effects of each mechanism are different. Fluid shifts are often cited as important factors in the pathophysiology of SANS (Stenger and Tarver, 2017; Lee et al., 2020), but gravitational loading may also play a role in SANS ocular findings. The removal of axial hydrostatic gradients was recently documented to decrease cardiac mass in an extreme duration swimmer, who spent 9–17 h per day in the supine or prone posture, and a long duration spaceflight astronaut, adding evidence that gravitational loading is important to consider in SANS pathology (MacNamara et al., 2021). Also, the loss in hydrostatic gradients that occur in space may create a pressure environment conducive to developing SANS (Buckey et al., 2022). Table 3 indicates the anticipated effects of fluid shift, tissue weight, and hydrostatic gradients for the conditions in this study in addition to head-down tilt (HDT) and microgravity. These anticipated effects can help hypothesize the source of difference between spaceflight analogue studies and long duration spaceflight studies.

**TABLE 3:** Anticipated effects of three proposed mechanisms during experimental conditions, HDT, and spaceflight.

Condition	Fluid shift	Tissue weight	Hydrostatic gradients
Seated Baseline	Baseline	Baseline	Baseline
Supine	Headward	Posterior	Posterior
Prone	Headward	Anterior	Anterior
LBPP	Headward	No effect	No effect
LBNP	Footward	No effect	No effect
HDT	Headward	Angled	Angled
Microgravity	Headward	Unloaded	Unloaded

In this study, IOP was affected by both fluid shifts and posture. While the mechanisms of these changes cannot be determined by causal relationship, our findings are consistent with that of the literature. Acute fluid shift effects on IOP and choroidal thickness observed in this study agree with previous observations (Taibbi et al., 2014; Macias et al., 2015; Anderson et al., 2017; Roberts et al., 2017). A previous study documented an increase in IOP within seconds of changing from the seated to 10^◦^ HDT. (Mader et al., 1990) IOP is a product of episcleral venous pressure by reduced aqueous outflow when elevated. (Leith, 1963) Mader et al. assert that increases in IOP from episcleral venous pressure would take several minutes to occur. (Mader et al., 1990) Mader et al. hypothesize that choroidal engorgement, not increased episcleral pressure, causes this initial, immediate spike in IOP. (Mader et al., 1990) A sudden increase of only 20 µL to the choroid can cause an immediate increase in IOP of 20 mmHg (Smith and Lewis, 1985), therefore small fluctuations in choroidal volume can cause this initial spike in IOP. The choroid lacks autoregulation, therefore, a primary force limiting a sudden rise in choroidal volume is the increasing IOP, due to the interface and compliance of vessels and humor. Similarly, IOP measurements in microgravity are initially elevated compared to supine values, both immediately upon entering microgravity (Draeger et al., 1993; Anderson et al., 2016) and early in flight (Mader, 1991). The initial increase in IOP in microgravity is theorized to be the result of choroidal expansion and increased episcleral venous pressure from headward fluid shifts, with the same causal reasoning explained for the IOP increases in HDT (Mader et al., 1990; Mader et al., 1993). In support of the theory for the initial increase, our results show an effect for headward fluid shifts increasing both IOP and choroidal thickness.

Over longer timescales, IOP in HDT and microgravity differ. In HDT, IOP remains elevated over 48 h compared to seated measurements. This sustained effect is likely due to an increase in episcleral venous pressure. (Mader et al., 1990) Data from the Lifetime Surveillance of Astronaut Health, however, suggest IOP, which is initially elevated in microgravity, returns to pre-flight seated values at or before flight day 30^1,2^. This is inconsistent with HDT data, where IOP remains elevated compared to baseline over 70 days (Cromwell et al., 2014). The subsequent IOP decrease back to baseline in microgravity is theorized to be the result of compensatory aqueous volume decrease in response to choroidal engorgement (Mader et al., 1990). While we did observe IOP changes, the time scales in this study are not sufficient to study the mechanism that causes the return to baseline of IOP in long duration spaceflight.

The loss in hydrostatic gradients may also play a role in changes to IOP, and our findings indicate a statistical effect of posture, beyond what was found for fluid shifts independently. We observed an IOP increase from the supine to prone position as the direction of the gravitational force is changed, in agreement with a previous study (Anderson et al., 2016). For spaceflight, microgravity likely represents an intermediate point between the supine and prone conditions suggesting that the loss of hydrostatic gradients will lead to an increase in measured IOP over supine values. In addition to the effects of gravitational loading on IOP, it was also statistically related to axial length, aqueous depth, and RNFL thickness. This is also in agreement with previous observations (Taibbi et al., 2014; Macias et al., 2015; Anderson et al., 2017).

Gravitational loading effects on IOP, axial length, aqueous depth, and RNFL thickness also agreed with previous observations (Taibbi et al., 2014; Macias et al., 2015; Anderson et al., 2017). A previous study observed diurnal variations in IOP and axial length, however, IOP changes were not found to cause axial length changes. (Wilson et al., 2006). In spaceflight, axial length may be shortened by roughly 280–304 μm (Masterova et al., 2018) due to globe flattening and long-term structural remodeling of the eye. These shifts cause the hyperopic shifts observed in long duration spaceflight astronauts (Stenger and Tarver, 2017; Lee et al., 2020). On short timescales, choroidal expansion coupled with age-related presbyopia have also been hypothesized to contribute to the symptomatic hyperopic shift during spaceflight (Lee et al., 2017). Another possible explanation for the hyperopic shift in spaceflight--corneal refractive power changes due to atmospheric pressure and oxygen partial pressure changes--is unlikely (Mader and White, 1995; Winkle et al., 1998). We note that the optical biometry axial length measurements in this study include retinal thickness, while spaceflight ultrasound biometry axial length measurements terminate at the inner limiting membrane (Dong et al., 2018). No corneal thickness changes were observed in this study as would be expected on these timescales from our prior work (Anderson et al., 2017). RNFL thickness increased from baseline to both supine and prone in our study. These changes were similar to the differences to those found comparing pre- and post-flight long duration spaceflight astronauts and comparing low myopic subjects and control subjects in another terrestrial study (Dong et al., 2018; Patel et al., 2018). We hypothesize that this occurs due to edema, although LBNP was not sufficient to mitigate this posture-induced change. Long duration spaceflight and HDT both induce an increase in retinal thickness as well (Laurie et al., 2020). The timescales of RNFL thickness changes from bed rest and spaceflight cannot be compared to the acute changes in our study. Total retinal thickness increases more in spaceflight than in HDT bed rest, although the fact that astronauts exercise daily and bed rest subjects do not may be a confounding factor to fluid environment differences (Laurie et al., 2020).

We report that chamber pressure, and not posture, changed subfoveal choroidal thickness. Previous studies have shown that subfoveal choroidal thickness increases in HDT, while peripapillary choroidal thickness does not. Astronauts do experience an increase in peripapillary choroidal thickness (Laurie et al., 2020), contrasting the HDT findings, since both cohorts experience cephalad fluid shifts. (Laurie et al., 2020) This may be due to removal of hydrostatic gradients in microgravity compared to the gradients experienced by subjects that accompany fluid shift in bed rest. The unloading of tissue weight in microgravity may also contribute to choroidal expansion. (Laurie et al., 2020) The removal of tissue weight allows the choroid blood volume to increase even if choroid blood pressure remains constant.

Due to the timescales of this study, our results are indicative of the acute responses of ocular geometry and pressure and the relationships between these variables. Additionally, this study was not designed to differentiate between the effects of hydrostatic gradients and tissue weight, as both factors are modified simultaneously with the gravitational loading associated with postural changes. In this study, subject weight was correlated with MAP changes, consistent with tissue weight compressive forces induced by postural changes. Subject weight was not correlated with IOP changes. Previous studies have shown correlations between *changes* in body weight and *changes* in IOP (Lam et al., 2017; Khan et al., 2021), but to our knowledge IOP has not been correlated directly with subject weight (i.e., lower body weights correspond to lower IOP). Despite this, the acute exposure to the experimental conditions implemented in this study represent a way to understand mechanistic impacts of variables that may be critical to understand the initial response of the eye and cardiovascular system to the spaceflight environment. Future work should include the investigation of countermeasures to account for the effects of both fluid shifts and gravitational loading since countermeasure development efforts have primarily focused on fluid shifts. Ongoing research by the authors includes the investigation of the influence of the body and eye’s anatomy, as well as the effects of tissue weight.

## Conclusion

Through acute postural and lower body pressure changes, the effects of hydrostatic gradients and fluids shifts were assessed to determine their influence on the eye. Both fluid shifts and gravitational loading acutely changed the geometry and pressures of the eye. We confirmed our hypotheses that both posture and pressure affect IOP, but only posture affects axial length and aqueous depth. Contrary to our hypotheses, fluid shifts effected choroid thickness instead of posture, and tissue weight did not affect IOP or MAP. Fluid shifts are often cited as important factors in the pathophysiology of SANS, but these data suggest gravitational loading may also play an important role in these ocular findings. Both of these factors should continue to be investigated as mechanisms relevant to understanding the etiology of SANS. The differences in the fluid environment and gravitational loading direction between spaceflight analog subjects such as bedrest and long duration spaceflight participants may contribute to explaining the difference in ocular findings between populations.

## Data Availability

The original contributions presented in the study are included in the article/Supplementary Materials, further inquiries can be directed to the corresponding author.
